# Effect of pyruvate kinase gene deletion on the physiology of *Corynebacterium glutamicum* ATCC13032 under biotin-sufficient non-glutamate-producing conditions: Enhanced biomass production

**DOI:** 10.1016/j.meteno.2015.07.001

**Published:** 2015-07-03

**Authors:** Kazunori Sawada, Masaru Wada, Takuya Hagiwara, Susumu Zen-in, Keita Imai, Atsushi Yokota

**Affiliations:** Laboratory of Microbial Physiology, Research Faculty of Agriculture, Hokkaido University, Kita-9 Nishi-9, Kita-ku, Sapporo 060-8589, Japan

**Keywords:** *Corynebacterium glutamicum*, Pyruvate kinase, Biomass production, Phosphoenolpyruvate carboxylase, Phosphoenolpyruvate carboxykinase, Malate:quinone oxidoreductase

## Abstract

The effect of pyruvate kinase gene (*pyk*) deletion on the physiology of *Corynebacterium glutamicum* ATCC13032 was investigated under biotin-sufficient, non-glutamate-producing conditions. In a complex medium containing 100 g/L glucose, a defined *pyk* deletion mutant, strain D1, exhibited 35% enhancement in glucose consumption rate, 37% increased growth and a 57% reduction in respiration rate compared to the wild-type parent. Significant upregulation of phosphoenolpyruvate (PEP) carboxylase and downregulation of PEP carboxykinase activities were observed in the D1 mutant, which may have prevented over-accumulation of PEP caused by the *pyk* deletion. Moreover, we found a dramatic 63% reduction in the activity of malate:quinone oxidoreductase (MQO) in the D1 mutant. MQO, a TCA cycle enzyme that converts malate to oxaloacetate (OAA), constitutes a major primary gate to the respiratory chain in *C. glutamicum*, thus explaining the reduced respiration rate in the mutant. Additionally, pyruvate carboxylase gene expression was downregulated in the mutant. These changes seemed to prevent OAA over-accumulation caused by the activity changes of PEP carboxylase/PEP carboxykinase. Intrinsically the same alterations were observed in the cultures conducted in a minimal medium containing 20 g/L glucose. Despite these responses in the mutant, metabolic distortion caused by *pyk* deletion under non-glutamate-producing conditions required amelioration by increased biomass production, as metabolome analysis revealed increased intracellular concentrations of several precursor metabolites for building block formation associated with *pyk* deletion. These fermentation profiles and metabolic alterations observed in the mutant reverted completely to the wild-type phenotypes in the *pyk*-complemented strain, suggesting the observed metabolic changes were caused by the *pyk* deletion. These results demonstrated multilateral strategies to overcome metabolic disturbance caused by *pyk* deletion in this bacterium.

## Highlights

• The effect of *pyk*-deletion was investigated under non-glutamate-producing conditions.
• *Pyk*-deletion induced enhanced growth, glucose consumption, and reduced respiration.
• Metabolic changes that suppressed PEP/OAA over-accumulation led to enhanced growth.
• MQO was proposed as a key controller regulating OAA formation and respiration.

## 1 Introduction

*Corynebacterium glutamicum* is a Gram-positive, non-pathogenic, rod-shaped and biotin-auxotrophic bacterium that shows abundant growth under aerobic conditions. *C. glutamicum* is used widely for industrial production of amino acids such as glutamate and lysine (Kimura, 2005, Kelle et al., 2005). Use of this microorganism in the fermentation industry is gaining in importance as *C. glutamicum* can produce a wide variety of metabolites, such as organic acids (Wieschalka et al., 2012), amino acid derivatives (Kind et al., 2011, Krause et al., 2010), alcohols (Blombach et al., 2011) and bioplastics (Jo et al., 2007).

**Fig. 1:**
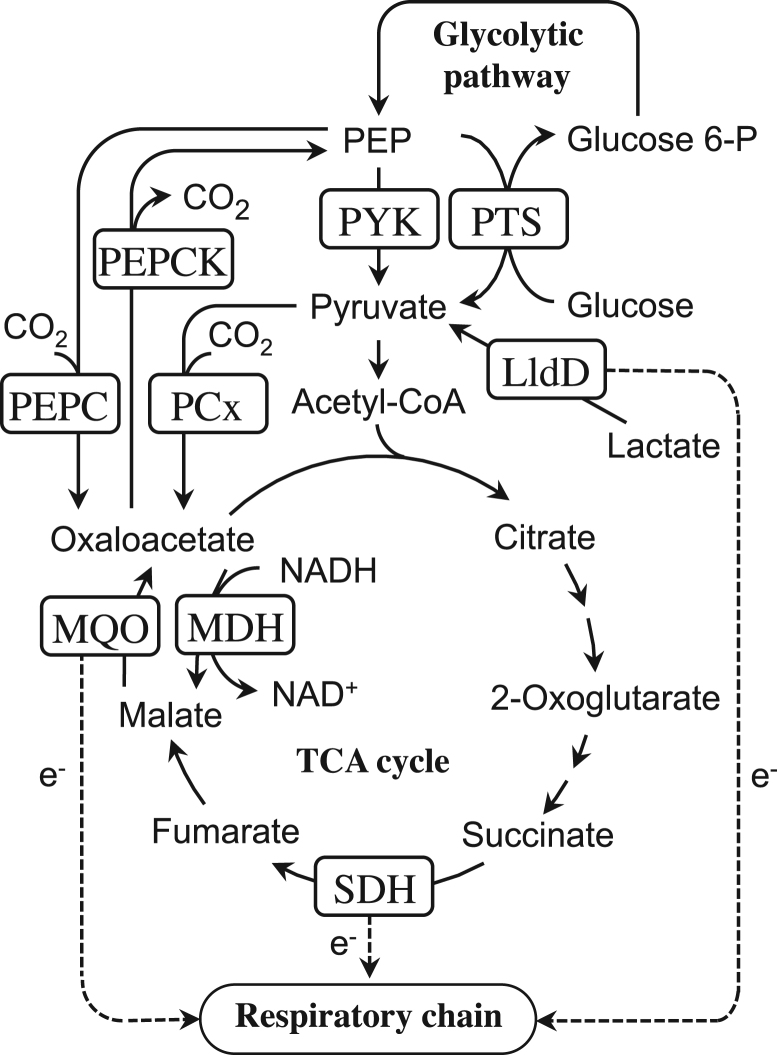
Central metabolic pathway around the phosphoenolpyruvate–oxaloacetate node in *Corynebacterium glutamicum.* Abbreviations: Glucose 6-P, glucose 6-phosphate; PEP, phosphoenolpyruvate; PTS, PEP: carbohydrate phosphotransferase system; PYK, pyruvate kinase; PEPC, phosphoenolpyruvate carboxylase; PEPCK, phosphoenolpyruvate carboxykinase; PCx, pyruvate carboxylase; LldD, quinone dependent L-lactate dehydrogenase; MQO, malate:quinone oxidoreductase; MDH, malate dehydrogenase; SDH, succinate dehydrogenase; e^−^, electron.

To improve the ability of metabolite production in this bacterium, pyruvate kinase (PYK) has long attracted attention for strain improvement, especially for lysine production. PYK of *C. glutamicum* catalyzes the conversion of phosphoenolpyruvate (PEP) to pyruvate ( Fig. 1), generating ATP from ADP. As the activity of this enzyme is allosterically regulated negatively by ATP and positively by AMP (Jetten et al., 1994, Ozaki and Shiio, 1969), PYK is a key enzyme that regulates glycolytic flux in response to the intracellular energy level. Furthermore, PYK is involved in the metabolism of PEP, an important intermediate that is then converted into oxaloacetate (OAA) by PEP carboxylase (PEPC) to replenish the carbon source in the TCA cycle (Fig. 1). OAA is a precursor for glutamic acid and aspartic acid family amino acids including lysine. Therefore, changing PYK activity may contribute to efficient metabolite production by this bacterium.

Previously, we reported the effect of *pyk* deletion on glucose metabolism in wild-type *C. glutamicum* ATCC13032 under glutamate-producing conditions induced by biotin limitation (Sawada et al., 2010). A defined *pyk*-deleted mutant (strain D1), derived using the double-crossover chromosome replacement technique, produced 25% more glutamate than the wild-type strain by altering the enzyme activities of the anaplerotic pathway to reduce PEP over-accumulation. This report was the first to demonstrate the primary effects of *pyk* deletion under biotin-limited conditions.

To our knowledge, no studies have investigated glucose metabolism in the simple *pyk*-deleted mutant under biotin-sufficient conditions. However, several reports have determined the effects of *pyk* deletion on lysine production under biotin-sufficient conditions using mutants derived by repeated random mutation and selection (Ozaki and Shiio, 1983, Shiio et al., 1987) or defined *pyk*-defective mutants (Becker et al., 2008, Gubler et al., 1994, Park et al., 1997). These reports provided insights into the effects of *pyk* deletion on glucose metabolism under biotin-sufficient lysine-producing conditions. However, it had been difficult to determine the primary effects of *pyk* deletion in a straightforward manner.

In this study, the primary effects of *pyk* deletion on glucose metabolism in *C. glutamicum* under biotin-sufficient conditions were evaluated using strain D1, a *C. glutamicum pyk*-deleted mutant (Sawada et al., 2010). The purpose of this study is not only to clarify metabolic alterations as the fundamental knowledge, but also to assess potentials of *pyk* deletion for metabolite production. In contrast to our previous study conducted under biotin-limited conditions (Sawada et al., 2010), strain D1 under biotin-sufficient conditions showed significantly increased biomass production and reduced respiration rate without glutamate production. Analyses of the metabolic alterations revealed enhanced anaplerotic activity that decreased the PEP over-accumulation caused by the *pyk* deletion. In addition, fine tuning activity of malate:quinone oxidoreductase (MQO), a respiratory chain component in *C. glutamicum* (Molenaar et al., 2000; Nantapong et al., 2004), seemed to preclude OAA over-accumulation. Enhanced biomass production may function to accommodate the precursor metabolites for building block formation formed differently in strain D1 due to *pyk* deletion during glucose metabolism. The results demonstrated unique strategies that relieve metabolic distortion caused by *pyk* deletion and highlighted the important roles played by MQO as a regulatory module for maintaining homeostasis in the central metabolism in *C. glutamicum*.

## 2 Materials and methods

### 2.1 Bacterial strains and media

*C. glutamicum* strains used in this study included the wild-type strain (ATCC 13032), a *pyk*-deleted mutant strain (D1), and a *pyk*-complemented strain (C1). The constructions of strains D1 and C1 were described previously (Sawada et al., 2010). For fermentation analysis, Medium 7 complete medium was used to refresh cultures (Sekine et al., 2001). To cultivate jar fermentor cultures in a complex medium, Medium S2 complex medium (Sekine et al., 2001) was used as a seed medium. Medium F4 complex medium (Sawada et al., 2010) containing 3 µg/L biotin or Medium F4 containing 60 µg/L biotin (termed Medium F5) was used as the main culture medium for biotin-limited and biotin-sufficient conditions, respectively. Medium F5 contained (per liter) 100 g glucose, 1 g KH_2_PO_4_, 1 g MgSO_4_·7H_2_O, 0.01 g FeSO_4_·7H_2_O, 0.01 g MnSO_4_·4–5H_2_O, 200 µg thiamine·HCl, 60 µg biotin, and 27.7 mL soybean-meal hydrolysate (total nitrogen, 35.0 g/L). Kanamycin (20 mg/L) was added to Medium 7 and Medium S2 when culturing strain C1 for preculture. To cultivate jar fermentor cultures in a minimal medium, Medium 7 was used as a first preculture medium, and optimized CGXII minimal medium (Keilhauer et al., 1993) containing (per liter) 40 g glucose, 20 g (NH_4_)_2_SO_4_, 5 g urea, 1 g KH_2_PO_4_, 0.25 g MgSO_4_·7H_2_O, 13.25 mg CaCl_2_·2H_2_O, 10 mg FeSO_4_·7H_2_O, 10 mg MnSO_4_·H_2_O, 1 mg ZnSO_4_·7H_2_O, 0.3 mg CuSO_4_·5H_2_O, 0.02 mg NiCl_2_·6H_2_O, 200 µg biotin, 30 mg protocatechuic acid, 100 µg thiamine·HCl was used as a second preculture medium. Salt and glucose solutions were autoclaved separately. Biotin, protocatechuic acid and thiamine·HCl solutions were filter-sterilized. The modified CGXII medium containing 20 g/L glucose, 10 g/L (NH_4_)_2_SO_4_ and no urea was used as the main culture medium. Kanamycin (20 mg/L) was added when culturing strain C1 for preculture.

### 2.2 Evaluation of fermentation profiles in Medium F4/F5

Culture procedures were followed as described previously (Sawada et al., 2010). Briefly, cells were refreshed on Medium 7 agar plates, and two successive precultures in Medium S2 were conducted to increase the culture volume. Cells were harvested in deceleration phase, washed twice and resuspended with 0.9% NaCl solution. The washed cells were grown in Medium F4 or Medium F5 to an initial optical density (OD)_660_ of 1.5. The main culture was grown in a 2-L jar fermentor (BMJ-02PI, ABLE Corporation, Tokyo, Japan) with a working volume of 1.2 L. The culture was aerated at 1 volume air·culture volume^−^^1^ min^−1^ (vvm) with stirring at 750 rpm. The pH was maintained at 7.0 with 28% (w/w) ammonia solution. All cultures were performed at 30 °C.

### 2.3 Evaluation of fermentation profiles in CGXII medium

Cells were refreshed on Medium 7 agar plates and two successive precultures were conducted. The first preculture was performed in 5 mL Medium 7 in a test tube for 10 h, and then the second preculture was conducted in 20 mL CGXII medium in 500 mL shaking flasks for 12 h, until the early stationary phase. Before inoculation, cells were harvested by centrifugation, washed twice with 0.9% NaCl and resuspended in the same solution. The washed cells were inoculated into CGXII medium to an initial OD_660_ of 0.1. The main culture was conducted in a 2-L jar fermentor with a working volume of 1.2 L. The culture was aerated at 1 vvm with stirring at 750 rpm. When the concentration of dissolved oxygen was less than 2 ppm, the stirring speed was increased to 980 rpm to maintain aerobic conditions. The pH was maintained at 7.0 with 3 N NaOH solution. All cultures were conducted at 30 °C.

### 2.4 Analytical methods

Growth, and residual glucose were analyzed as described previously (Sekine et al., 2001). The specific glucose consumption rate was defined as consumed glucose (g) per dry cell weight (g) per hour (Sawada et al., 2010). The dry cell weights of the wild-type, D1 and C1 strains cultured in Medium F5 were calculated from the values at 1 OD_660_ corresponding to 0.29, 0.25 and 0.28 g dry cell weight per liter, respectively. The dry cell weights of the wild-type, D1 and C1 strains cultured in GCXII were calculated from the values at 1 OD_660_ corresponding to 0.25, 0.24 and 0.25 g dry cell weight per liter, respectively. The level of dissolved oxygen in the culture broth was monitored using a dissolved oxygen electrode (ABLE Corp.). The specific respiration rate was measured using a dissolved oxygen analyzer (YSI 5300A Biological Oxygen Monitor; YSI Incorporated, Yellow Springs, OH, USA). The measurement was done in an air-tight camber at 30 °C using cells appropriately diluted with pre-warmed S2 medium (Sekine et al., 2001) after harvesting from the jar-fermentor. The oxygen solubility in the medium at 30 °C was assumed to be 0.220 mM. The specific respiration rate was defined as consumed O_2_ (μmol) per dry cell weight (mg) per minute. Organic acids in the culture broth were determined by high-performance liquid chromatography (HPLC) (AMINEX HPX-87H, Bio-Rad Laboratories, Inc., Hercules, CA, USA; mobile phase, 0.01 N H_2_SO_4_; flow rate, 0.6 mL/min; detection, absorbance at 210 nm), as described previously (Sekine et al., 2001).

### 2.5 Enzyme activity measurements

Enzyme activities were measured using cells cultured in a 2-L jar fermentor to deceleration phase, as described in previous sections. Cells were harvested by centrifugation, washed twice with 0.2% KCl solution, and stored at −80 °C until use. PYK, PEPC, PEP carboxykinase (PEPCK) (Sawada et al., 2010) and malate dehydrogenase (MDH) (Sawada et al., 2012) activities were measured using crude cell extract as the enzyme source. MQO, l-lactate dehydrogenase (LldD), succinate dehydrogenase (SDH) and NADH dehydrogenase (NDH-II) activities were measured using a membrane fraction suspension as described previously (Sawada et al., 2012). Protein concentrations of crude cell extracts for use in the PYK, PEPC, PEPCK and MDH assays were determined using a Bio-Rad Protein Assay Kit (Bio-Rad Laboratories, Inc.), while that of the membrane fragment suspensions for MQO, LldD, SDH and NDH-II assays were measured with a Bio-Rad DC Protein Assay Kit (Bio-Rad Laboratories, Inc.); both assays used bovine serum albumin as the standard. The specific activity of each enzyme was expressed as nmol per minute per mg protein. All enzyme activities were assayed at 25 °C.

### 2.6 Quantitative real-time PCR analysis

To analyze the genes encoding PEPC (*ppc*), PEPCK (*pck*), pyruvate carboxylase (PCx, *pyc*) and MQO (*mqo*), total RNA extraction from cells at early stationary phase, cDNA synthesis and quantitative real-time PCR using the LUX detection system (Invitrogen; Life Technologies Corporation, Carlsbad, CA, USA) were performed as described previously (Sawada et al., 2010).

To analyze genes encoding LldD (*lldD*), SDH (*sdhA*) and NDH-II (*ndh*), cells were cultured in a 2-L jar fermentor to deceleration phase, and two volumes of RNAprotect Bacteria Reagent (QIAGEN, Hilden, Germany) were added, incubated for 5 min and centrifuged at 6000×*g* for 5 min at room temperature. The supernatant was removed and the cell pellet was stored at −80 °C until use. Total RNA was extracted with the RNeasy Mini Kit (Qiagen). The cell pellet was resuspended in 0.7-mL RLT buffer containing β-mercaptoethanol (Qiagen). The cell suspension was mixed with 0.5 g of zirconia beads (diameter 0.1 mm), and then disrupted by vigorous shaking at 2000 rpm in Multi-beads shocker (Yasui Kikai Corporation, Osaka, Japan) at 4 °C for 360 s with a 15-s break after each 45 s run. The resulting mixture was centrifuged at 15,000 rpm. The supernatant was processed using the RNeasy system (Qiagen) according to the manufacturer's instructions. Trace amounts of genomic DNA were removed by DNase I treatment (Ambion; Life Technologies Corporation) following the manufacturer's instructions, and cDNA synthesis was performed as described previously (Sawada et al., 2010). Quantitative real-time PCR was carried out using Power SYBR Green PCR Master Mix (Applied Biosystems; Life Technologies Corporation) according to the manufacturer's instructions. The sequences of the forward and reverse primers for the *pyc*, *mqo*, *lldD*, *sdhA* and *ndh* target genes are listed in Table 1. The primer sequences for the *ppc* and *pck* genes were described previously (Sawada et al., 2010). Each sample was analyzed at least in duplicate. The 16S rRNA gene was used as an endogenous control. A relative standard curve method was used to calculate the relative expression level of the target genes.

**Table 1:** Sequences of primers used in real-time PCR.

Primer name	Sequence
*LUX detection system*	
*pyc*-f	CGGTGGTATCCAGGTTGAGCACAC^⁎^G
*pyc*-r	ATCTGCGCCTTCACCAGGTC
*mqo*-f	CGGCGGAGGACGACTCTTGTGC^⁎^G
*mqo*-r	AGCCAAGCTGGACTCAGATCG
16S rRNA-f	CGGGTGAGATGTTGGGTTAAGTCC^⁎^G
16S rRNA-r	CACAATGTGCTGGCAACATAAGA
*Power SYBR Green PCR Master Mix*	
*lldD*-f	GATTCCCGCAACGGATTCTC
*lldD*-r	AGGAAAGGGATGCGAACTCA
*sdhA*-f	CCATCATGCGTGCATACGAA
*sdhA*-r	AGAATGGTCTTGGACTGCCA
*ndh*-f	ACCTACAAGACCAAGGACGG
*ndh*-r	GCGGACCAAATCTTGCAGAA
16S rRNA-SYBR-f	CCTGGTGTAGCGGTGAAATG
16S rRNA-SYBR-r	CGTCAGTTACTGCCCAGAGA

^⁎^ 6-Carboxy-fluorescein (FAM).

### 2.7 Metabolome analysis

Cells cultured in CGXII medium were harvested in both the logarithmic phase (6 h) and deceleration phase (11 h). The metabolites in the cells were extracted as described previously (Bolten et al., 2007). Metabolite concentrations in the cells were measured using capillary electrophoresis and time-of flight mass spectrometry (CE-TOFMS) technique (Ohashi et al., 2008).

## 3 Results

### 3.1 Effect of *pyk* deletion on glucose metabolism under biotin-sufficient conditions in Medium F5

**Fig. 2:**
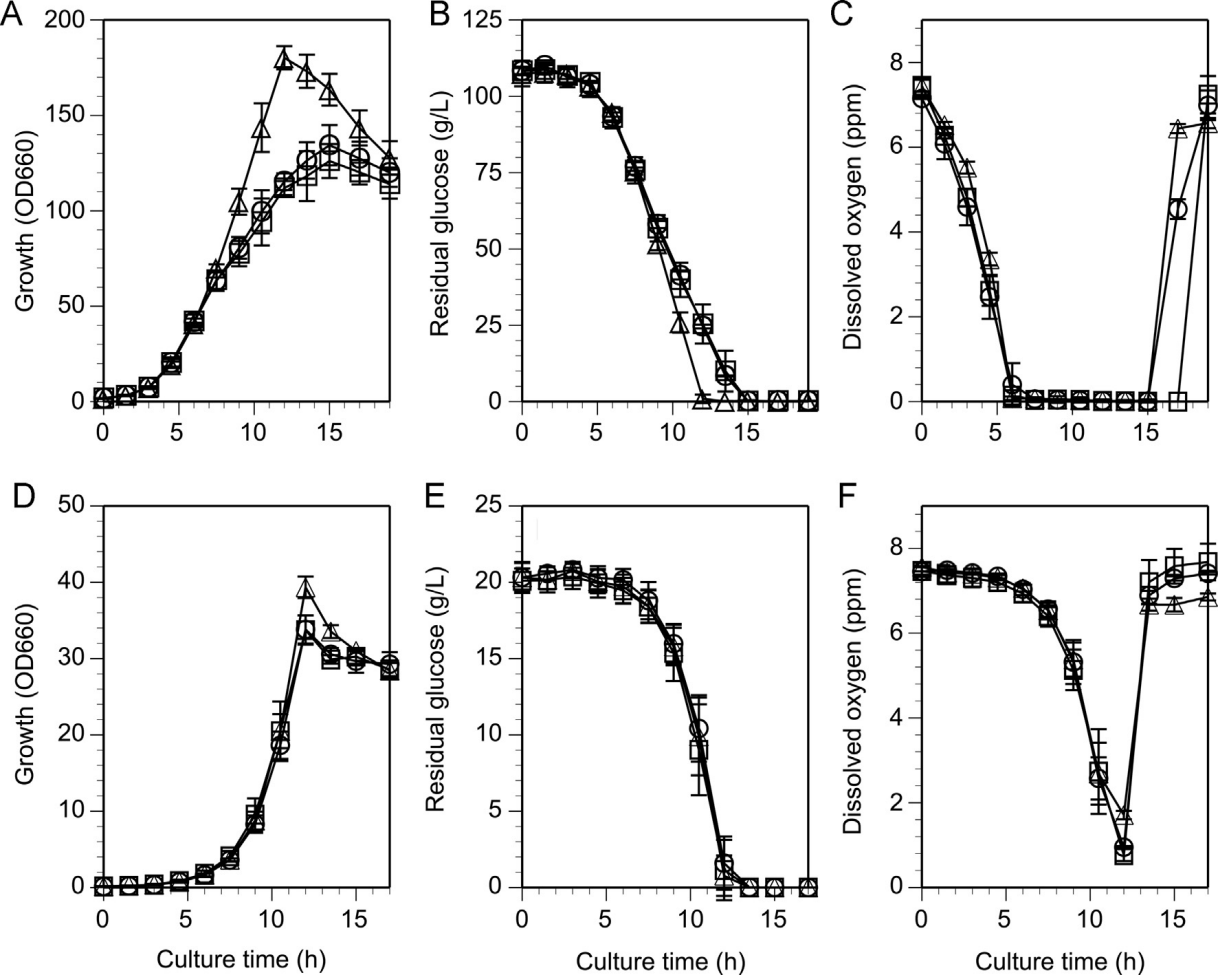
Profiles in batch cultures of the wild-type, *pyk*-deleted mutant (D1) and *pyk*-complemented (C1) strains cultured in Medium F5 (A, B, C) and CGXII medium (D, E, F). Each strain was cultured in a 2-L jar fermentor under biotin-sufficient conditions. A, D: Growth, B, E: Residual glucose, C, F: Dissolved oxygen. Symbols: circle, wild-type strain; triangle, D1 strain; square, C1 strain. Values are means of three independent experiments. Bars represent the standard deviations.

To investigate the effect of *pyk* deletion on fermentation profiles under biotin-sufficient conditions, the wild-type, D1 and C1 strains were cultured in 2-L jar fermentors with Medium F5, a complex medium for metabolite production containing 100 g/L glucose. Although the specific growth rates were similar, the maximum growth of strain D1 was about 37% higher than that of the wild-type or C1 strains (Table 2 and Fig. 2A), as determined using OD_660_. To confirm that the increased growth actually represented increased biomass production, the dry cell weight of each strain was measured. The calculated maximum biomass production of strain D1 in dry cell weight was 18% higher than that of the wild-type and C1 strains (Table 2). The apparent glucose consumption of strain D1 was faster than that of the wild-type strain (Fig. 2B). The calculated specific glucose consumption rate of strain D1 was 35% higher than that of the wild-type during deceleration phase (Table 2). Dissolved oxygen was depleted in cultures of all strains during the deceleration phase (Fig. 2C). The three strains produced a considerable amount of lactic acid (wild-type strain, 7.0 g/L; strain D1, 11.2 g/L; strain C1, 7.6 g/L), and a small amount of succinic acid (wild-type strain, 1.1 g/L; strain D1, 4.1 g/L; strain C1, 0.2 g/L), which are produced in the presence of insufficient oxygen supply (Fukui et al., 2011). No other metabolites of organic acids and amino acids were detected in meaningful amounts, with the exception of acetate (wild-type strain, 5.6 g/L; strain D1, 3.8 g/L; strain C1, 5.8 g/L). The specific respiration rate was assayed in deceleration phase cells (Table 2). Interestingly, strain D1 showed 57% lower specific respiration rate than that of the wild-type. To check if this was also the case under biotin-limited conditions, we have measured the respiration rates using cells cultured in Medium F4 under biotin-limited glutamic-acid producing conditions used in our previous study (Sawada et al., 2010). The results showed comparable respiration rates between the two strains (Table 2), suggesting respiration control depending on biotin concentration. All C1 strain profiles were similar to those of the wild-type strain, indicating that the differences between the wild-type and D1 strains were caused by the *pyk* deletion (Table 2).

**Table 2:** Fermentation parameters of the jar fermentor cultures.

Fermentation parameter in different culture medium	Value of each strain			Ratio
	Wild-type	Strain D1	Strain C1	(D1/Wild-type)
*Medium F5*				
Specific growth rate (h^−^^1^)	0.57±0.01	0.58±0.02	0.58±0.01	1.02
Maximum growth (OD_660_)	128.4±5.9	176.3±6.6	119.9±5.5	1.37†
Dry cell weight (g/L)	37.4±3.5	44.1±0.6	34.1±1.8	1.18†
Glucose consumption rate^a^	0.40±0.03	0.54±0.03	0.45±0.02	1.35†
Specific respiration rate^b^	0.30±0.03	0.13±0.003	0.31±0.04	0.433†
*Medium F4*				
Specific respiration rate^b^	0.16±0.01	0.16±0.026	0.16±0.01	1.00
*CGXII medium*				
Specific growth rate (h^−1^)	0.54±0.01	0.56±0.02	0.55±0.01	1.04
Maximum growth (OD_660_)	33.8±1.8	39.3±1.4	33.7±1.9	1.16†
Dry cell weight (g/L)	8.4±0.6	9.3±0.2	8.4±0.6	1.11†
Glucose consumption rate^a^	1.02±0.03	1.00±0.05	0.86±0.12	0.980

Values are mean±SD (*n*=3). Specific growth rates were calculated from data obtained in logarithmic phase (Medium F5, between 3 and 6 h; CGXII medium, between 6 and 9 h). Glucose consumption rates were calculated from data obtained in deceleration phase (Medium F5, between 9 and 10.5 h; CGXII medium, between 10.5 and 12 h). Specific respiration rates were measured with cells obtained in stationary phase (11 h).

† Significant *t*-test differences (*P*<0.05).

a Glucose (g)·[dry cell weight (g)]^−1^ h^−^^1^.

b μmol O_2_·[dry cell weight (mg)]^−^^1^ min^−^^1^.

### 3.2 Effect of *pyk* deletion on glucose metabolism under biotin-sufficient conditions in CGXII medium

To conduct physiologically more precise analysis of the fermentation characteristics of strain D1, these strains were cultured in a minimal medium CGXII containing 20 g/L glucose in 2-L jar fermentors. The advantages of using these culture conditions were that all the building blocks need to be synthesized *de novo* and that the dissolved oxygen was not depleted during the cultures due to the lower biomass production than that in Medium F5. Corresponding to the results obtained from Medium F5 cultures, the similar growth rates between the wild-type and D1 strains and the higher maximum growth in strain D1 than the wild-type strain by 1.2-fold in OD_660_ basis and 1.1-fold in dry cell weight basis were observed (Table 2 and Fig. 2D). These differences were statistically significant (Table 2). However, in contrast to the results obtained in Medium F5 cultures, enhanced glucose consumption was not observed in strain D1 in comparison to the wild-type strain (Table 2 and Fig. 2E). The dissolved oxygen was not depleted during the cultures (Fig. 2F). Although respiration rate was not measured, the decrease in dissolved oxygen concentration of strain D1 was the most moderate among these strains implying the reduced respiration rate in strain D1 as observed in Medium F5 culture. Only trace amounts of lactate (wild-type strain, 0.18 g/L; strain D1, 0.23 g/L; strain C1, 0.14 g/L) were detected in the culture supernatants. No other metabolites of organic acids and amino acids were detected in meaningful amounts (less than 0.1 g/L). These results were in contrast to those obtained in Medium 5, indicating efficient oxidation of glucose under aerobic conditions. All C1 strain profiles were similar to those of the wild-type strain (Table 2).

### 3.3 Enzyme activity measurements

To investigate further the alterations of glucose metabolism, enhancement of biomass production and decrease in respiratory activity caused by the *pyk* deletion, the activities of the enzymes involved in PEP and OAA metabolism were measured, because the imbalanced formation of these metabolites seemed to cause metabolic distortion in the *pyk*-deleted mutant, as in the case of biotin-limited conditions (Sawada et al., 2010). The measurements were conducted using cells harvested from cultures in Medium F5 or CGXII at deceleration phase and the results are summarized in Table 3.

**Table 3:** Activities of enzymes involved in the phosphoenolpyruvate/oxaloacetate metabolism and the electron transfer to respiratory chain.

Enzyme	Specific activity [nmol min^–1^ (mg protein)^–1^]			Ratio (D1/Wild-type)
	Wild-type	Strain D1	Strain C1	
*Medium F5*				
PYK	931±197	<10	1057±199	–
PEPC	103±7.6	157±7.9	136±33	1.52†
PEPCK	186±14	22.5± 2.9	156± 5.0	0.121†
MQO	698±99	260±39	770±75	0.372†
MDH	1743±360	1713±162	1684±236	0.983
LldD	61.5±14	35.4±17	53.9±22	0.576
SDH	64.0±6.0	53.7±16	53.8±8.7	0.839
NDH-II	451±35	252±44	467±10	0.559†

*Medium F4*				
MQO	384±55	417±89	303±63	1.09

*CGXII medium*				
PEPC	98.7±11.0	102±13	95.2±10.0	1.03
PEPCK	35.1±5.1	12.4± 0.9	39.3± 1.4	0.353†
MQO	259±20	151±34	250±37	0.583†

Values are mean±SD (*n*=3).

† Significant *t*-test differences (*P*<0.05).

When the measurements were conducted using cells cultured in Medium F5, PYK activity in strain D1 was below the detection limit [<10 nmol min^−^^1^·(mg protein)^−1^]. This result verified that both the *pyk* gene and PYK activity were completely eliminated in strain D1. Furthermore, nearly identical PYK activity levels were observed in C1 and wild-type strains, indicating the successful restoration of PYK activity. Similar to observations under biotin-limited conditions (Sawada et al., 2010), strain D1 increased PEPC activity by 52% and decreased PEPCK activity by 88% compared to the wild-type strain. The coordinated change of PEPC and PEPCK activities may be an important strategy to avoid PEP over-accumulation caused by the *pyk* deletion (Fig. 1); this change seemed independent of the biotin concentration, and was considered a trait intrinsic to this *pyk*-deleted mutant. Furthermore, strain D1 significantly decreased MQO activity by 63% compared to the wild-type strain. MQO is known to constitute a coupling reaction with MDH for NADH reoxidization; MQO catalyzes the conversion of malate to OAA in the TCA cycle, with electron transfer to the respiratory chain using menaquinone as the electron acceptor; MDH reoxidizes NADH by converting OAA to malate (Molenaar et al., 2000, Nantapong et al., 2004) (Fig. 1). On the other hand, no activity change was observed in MDH in strain D1. Since MDH catalyzes the reverse reaction of the TCA cycle from OAA to malate (Molenaar et al., 2000), the reduced MQO activity may relieve the over-accumulation of OAA, possibly derived from the coordinated changes of PEPC and PEPCK activity. On the other hand, in a separate experiment, comparable MQO activity was observed between wild-type and D1 strains when measured with cells cultured in Medium F4 under biotin-limited glutamate-producing conditions used in our previous study (Sawada et al., 2010) (Table 3). Decreased activity of LldD, SDH and NDH-II were found in strain D1, which also transferred electrons using menaquinone as the electron acceptor to the respiratory chain (Bott and Niebisch, 2003) (Fig. 1). Thus, decreased MQO, LldD, SDH and NDH-II activities seemed to contribute to the decreased specific respiration rate in strain D1. Pyruvate carboxylase (PCx) is an important enzyme involved in OAA metabolism (Fig. 1), as PCx functions as the main anaplerotic enzyme under biotin-sufficient conditions (Petersen et al., 2000). However, we were unable to measure the activity of PCx by the method reported previously (Peters-Wendisch et al., 1997) due to technical difficulties. The specific activities of all these enzymes in strain C1 were similar to those of the wild-type strain (Table 3), indicating that these changes were caused by the *pyk* deletion.

Similar results were obtained when PEPC, PEPCK and MQO activities were measured with cells cultured in CGXII medium, although no increase in PEPC and relatively moderate fold differences were observed in PEPCK and MQO activities as compared to those observed with cells cultured in Medium F5. Probably these differences reflected severer metabolic distortion in Medium F5 cultures than that in CGXII cultures.

### 3.4 Transcriptional analysis

As the specific activities of the enzymes involved in PEP and OAA metabolism were altered significantly, the transcriptional levels of *ppc*, *pck*, *mqo*, *lldD*, *sdhA* and *ndh* of cells cultured in Medium F5 were measured by quantitative real-time PCR analysis. In addition, the level of *pyc* expression was determined. As shown in Table 4, significant reductions in the transcriptional levels of *pck*, *mqo*, *lldD* and *sdhA* were detected in strain D1 compared to the wild-type strain, suggesting that decreased PEPCK and MQO activities resulted from decreased gene expression. On the other hand, comparable transcriptional levels of *mqo* were observed between wild-type and D1 strains when measured with cells cultured under biotin-limited glutamate-producing conditions in Medium F4 used in our previous study (Sawada et al., 2010), demonstrating a good agreement with MQO activities in these cells samples (Table 3). Transcriptional levels of *pyc* (PCx gene) in strain D1 were reduced significantly by 70% compared to the wild-type strain (Table 4). These results indicated that strain D1 may have decreased PCx activity. Several global transcriptional regulators such as RamA (for *pck*, *mqo*, *lldD* and *sdhA*), RamB (*sdhA* and *pyc)* and GlxR (*lldD* and *sdhA*) have been suggested to control the expression of the respective genes (Auchter et al., 2011, Teramoto et al., 2011, Klaffl et al., 2013). However, the relationship between these regulators and the altered transcriptional levels of these genes remains unclear. In contrast to the specific activity of NDH-II, the transcriptional level of *ndh* was increased significantly in strain D1 compared to the wild-type strain. A reasonable explanation for this contradiction was not found in this study.

**Table 4:** mRNA levels of genes involved in phosphoenolpyruvate/oxaloacetate metabolism and the electron transfer to respiratory chain relative to 16S rRNA.

Gene	Relative quantity of mRNA^a^		Ratio (D1/Wild-type)
	Wild-type	Strain D1	
*Medium F5*			
*ppc*	2.57±1.11	2.71±0.94	1.05
*pck*	11.7±1.54	1.54±0.37	0.132†
*pyc*	1.47±0.34	0.44±0.07	0.299†
*mqo*	2.59±0.38	1.58±0.07	0.610†
*lldD*	0.59±0.11	0.19±0.01	0.322†
*sdhA*	11.0±1.88	4.50±0.56	0.409†
*ndh*	0.80±0.17	1.92±0.13	2.40†

*Medium F4*			
*mqo*	0.38±0.19	0.34±0.27	0.895

*CGXII medium*			
*ppc*	2.54± 0.59	2.67±0.62	1.05
*pck*	3.51±0.25	2.43±0.25	0.692†
*pyc*	5.73±0.93	3.89±0.65	0.679†
*mqo*	7.02±2.76	4.53±1.56	0.645†

Values are mean±SD (*n*=3) on a 10^–4^ scale.

a The transcriptional level of 16S rRNA was taken as 1.0.

† Significant *t*-test differences (*P*<0.05).

The transcriptional levels of the selected enzyme genes, namely, *ppc*, *pck*, *mqo* and *pyc* were also measured with cells cultured in CGXII medium. The results showed significant reductions in the transcriptional levels in *pck*, *mqo* and *pyc* in strain D1. These results were comparable to those observed with cells cultured in Medium F5. However, the fold differences were again much moderate than those observed in Medium F5 cultures, suggesting severer metabolic distortion in Medium F5 cultures than in CGXII cultures.

### 3.5 Metabolome analysis

To understand metabolic alterations associated with *pyk* deletion, metabolome analysis was conducted to overview metabolite distribution. Samples were prepared from cells cultured in CGXII medium because of the following two reasons; i) physiologically more precise without both the depletion of dissolved oxygen during the culture and the salvage synthesis of the intermediate metabolites for biomass production, ii) principally the same metabolic characteristics as those observed in Medium F5 cultures (Tables 2–4 and Fig. 2). Samples were taken from the cultures at 6 h and 11 h in CGXII medium. The important results were summarized in Table 5 (ratio of D1 to wild-type strains) and the overall results were presented in Table S1 (concentration and the ratio). Metabolome analysis revealed imbalanced distribution of intermediate metabolites in strain D1. The *pyk* deletion provoked accumulation of many intermediate metabolites upstream of PYK reaction. They included several precursor metabolites in glycolytic pathway and pentose phosphate pathway for biomass production such as PEP, 3-phosphoglyceric acid, dihydroxyaceton phosphate, fructose 6-phosphate, glucose 6-phosphate and ribose 5-phosphate. In contrast, intermediate metabolites downstream of PYK reaction including several TCA cycle intermediates were decreased, although OAA was not detected by the analytical system employed. They included several precursor metabolites such as pyruvate, acetyl CoA and 2-oxoglutaric acid. These results clearly indicated that the absence of PYK reaction constituted a major bottleneck in the carbon flow in the central metabolism in strain D1. In general the intermediate concentration ratios of D1 to wild-type strains amplified overtime (Table 5, from 6 h to 11 h), suggesting an increased metabolic distortion toward deceleration phase culture in strain D1. The metabolite concentrations in strain C1 were similar to those of the wild-type strain (Table S1), indicating that these changes were caused by the *pyk* deletion.

**Table 5:** Ratios of metabolite concentration in D1 strain cells to that in wild-type strain cells cultured in CGXII medium.

Metabolite	Ratio	
	(D6 h/W6 h)	(D11 h/W11 h)
**Glycolytic pathway**		
Glucose 6-phosphate^a^	2.63	2.59
Fructose 6-phosphate^a^	2.28	1.79
Fructose 1,6-diphosphate	1.58	1.41
Dihydroxyacetone phosphate^a^	1.22	1.70
3-Phosphoglyceric acid^a^	1.93	3.27
PEP^a^	2.11	5.59
Pyruvic acid^a^	0.899	0.214

**TCA cycle**		
Acetyl CoA^a^	0.663	0.542
Citric acid	0.523	0.676
2-Oxoglutaric acid^a^	1.17	0.816
Succinic acid	0.708	0.522
Fumaric acid	1.03	0.719
Malic acid	0.844	0.656

**Pentose phosphate pathway**		
6-Phosphogluconic acid	1.83	8.23
Ribulose 5-phosphate	1.79	4.32
Ribose 5-phosphate^a^	1.55	2.57
Sedoheptulose 7-phosphate	2.51	6.31

Concentration data from Table S1 were used for calculation. D6 h/W6 h, ratio of metabolite concentration of strain D1 cultured for 6 h to that of wild-type strain cultured for 6 h. D11 h/W11 h, ratio of metabolite concentration of strain D1 cultured for 11 h to that of wild-type strain cultured for 11 h.

a Precursor metabolites essential for biomass synthesis.

## 4 Discussion

**Fig. 3:**
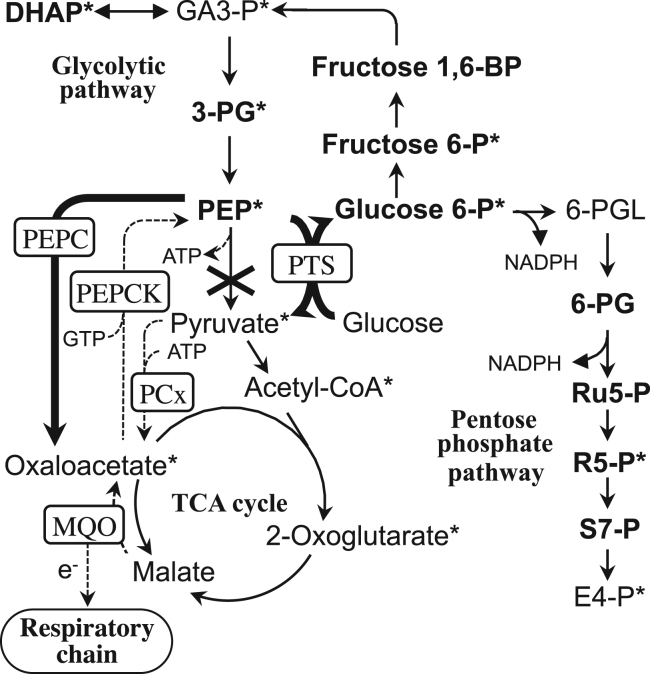
Schematic model of carbon flux in strain D1. Cross represents the *pyk* deletion. Thick arrow line indicates increased flux, and the dotted arrow line indicates decreased flux deduced from enzyme activity measurement, transcriptional analysis or rate analysis. Increased metabolites detected in metabolome analysis in strain D1 are shown in boldface. Asterisk indicates a precursor metabolite for biomass synthesis. Abbreviations: Fructose 6-P, fructose 6-phosphate; Fructose 1,6-BP, fructose 1,6-bisphosphate; GA3-P, glyceraldehyde 3-phosphate; DHAP, dihydroxyacetone phosphate; 3-PG, glycerate 3- phosphate; 6-PGL, 6-phosphogluconolactone; 6-PG, 6-phosphogluconate; Ru5-P, ribulose 5-phosphate; R5-P, ribose 5-phosphate; S7-P, sedoheptulose 7-phosphate; E4-P, erythrose 4-phosphate. Other abbreviations are the same as shown in the legend to Fig. 1.

This study investigated the primary effect of *pyk* knockout on the glucose metabolism of *C. glutamicum*, an industrially important actinobacterium for metabolite production, under biotin-sufficient non-glutamate-producing conditions. A defined *C. glutamicum pyk*-deleted mutant, strain D1, showed significantly increased biomass production (by 37% at OD_660_ level) (Fig. 2A, Table 2) compared to the wild-type strain in Medium F5, which was in contrast to our previous results that the same stain showed decreased biomass production (15% decrease) with significantly increased glutamate production (25% increase) compared to the wild-type strain under biotin-limited glutamate-producing conditions in Medium F4 (the same composition as Medium F5 except biotin concentration was limited to 3 µg/L) (Sawada et al., 2010). In both cases, the glucose consumption rates were increased by about 30% at deceleration phase culture, while no difference was observed in the growth rate between the wild-type and D1 strains (Table 2, Sawada et al., 2010). Interestingly, a 57% decrease in respiration rate was observed in strain D1 in Medium F5 cultures, which was not observed under the biotin-limited conditions in Medium F4 cultures (Table 2). Increased growth level was also observed in the minimal medium CGXII (Fig. 2D, Table 2). The overall fermentation profiles implied different strategies to manage metabolic stresses caused by the *pyk* deletion according to the biotin concentrations. Besides these fermentation profiles, the metabolome analysis demonstrated imbalanced distribution of intermediate metabolites in strain D1 compared to the wild-type starin; accumulation of intermediates in glycolytic and pentose phosphate pathways upstream of PYK reaction, especially PEP, and the decrease of those downstream of PYK reaction, although OAA was not detected in the analytical system employed (Table 5 and S1, Fig. 3).

As the mechanism to relieve PEP over-accumulation, the coordinated change of PEPC and PEPCK expression, *i.e.*, up-regulation of PEPC (in Medium F5 only) and down-regulation of PEPCK (in both Medium F5 and CGXII) at both the enzyme activity and transcriptional levels in strain D1 compared to the wild-type starin was identified (Tables 3 and 4). As similar changes were also observed in our previous study conducted under biotin-limited conditions (Sawada et al., 2010), these coordinated alterations might constitute a primary or common response independent from biotin concentrations to relieve PEP over-accumulation by enhancing flux from PEP to OAA in the *pyk*-deleted mutant.

Another common strategy to relieve PEP over-accumulation seemed the enhanced glucose consumption in strain D1, as *C. glutamicum* takes up glucose mainly *via* the PEP: carbohydrate phosphotransferase system (PTS) with concomitant conversion of PEP to pyruvate (Ikeda, 2012) (Fig. 1, Fig. 3). It has been reported that increased PEP/pyruvate ratio causes activation of PTS (Patnaik et al., 1992). From the metabolome data (Table S1), the PEP/pyruvate ratios after 11 h culture were calculated to be 0.847 and 22.1 in wild-type strain and D1 mutant, respectively. Thus, these values may explain the enhanced glucose consumption rate in strain D1. The enhanced glucose uptake by PTS also seemed to be beneficial for supplying pyruvate the intracellular concentration of which dramatically decreased in strain D1 after the culture for 11 h (Table S1). Alleviation of PEP over-accumulation by the enhanced PTS reaction inevitably reinforces over-accumulation of glycolysis and pentose phosphate pathway intermediates upstream of PEP, which, however, must be less toxic than PEP stress.

Although not able to be monitored in metabolome analysis, OAA over-accumulation was deduced as the result of coordinated alterations in PEPC and PEPCK activities to enhance flux from PEP to OAA in strain D1. It is probable that reduced gene expression in PCx may be a response to alleviate OAA over-accumulation in strain D1 (Table 4). The reduced MQO activities in Medium F5 and CGXII cultures (Table 3) can also be interpreted to preclude OAA over-accumulation in strain D1 as MQO plays a major role in converting malate to OAA in *C. glutamicum* (Molenaar et al., 1998, Molenaar et al., 2000). Interestingly, the reduction of MQO activity in strain D1 was not observed in the biotin-limited Medium F4 cultures (Table 3), where glutamate production takes place. This may reflect a difference in OAA metabolism between biotin-limited and -sufficient conditions. It is probable that glutamate production itself may relieve OAA over-accumulation as the TCA cycle intermediates are drained off to glutamate; however, they are not removed under biotin-sufficient conditions, and thus, the metabolic disturbance caused by *pyk* deletion probably becomes much severer than under biotin-limited conditions. Therefore, fine-tuning of MQO activity is important as a secondary measure to relieve metabolic distortion around the OAA node in *C. glutamicum*.

However, the final amelioration of metabolic distortion seemed to be completed by the conversion of excess precursor metabolites into biomass during deceleration phase (Fig. 3). Metabolome analysis revealed imbalanced distribution of intermediate metabolites in strain D1 associated with the *pyk* deletion (Tables 5 and S1). The variation included accumulation of several precursor metabolites in glycolytic and pentose phosphate pathways for biomass production. In general the intermediate concentration ratios of D1 to wild-type strains amplified overtime (from 6 h to 11 h), suggesting an increased metabolic distortion toward deceleration phase culture in strain D1. Besides precursor metabolites, biomass production requires both the reducing equivalent, NADPH, and the biological energy, ATP. It was assumed that availability of NADPH was higher in strain D1 than the wild-type strain as the concentrations of pentose phosphate pathway intermediate such as glucose 6-phosphate, 6-phosphogluconate and ribulose 5-phosphate, involved in NADPH generating reaction were over-accumulated in strain D1 (Fig. 3, Table 5). It has been reported that PYK (ATP generating enzyme), PEPCK (GTP consuming enzyme) and PCx (ATP consuming enzyme) constitute an energy consuming futile cycle (Fig. 3) (Petersen et al., 2000). The *pyk* deletion stops operation of this futile cycle. Furthermore, the reduction of PEPCK activities (Table 3) and the possible reduction of PCx activities (Table 4) may lead to saving of the biological energy, *i.e.*, ATP/GTP. Although the precise mechanisms underlying increased biomass production in strain D1 remained unclear, it seems rational to take all these factors into consideration.

Here, important roles played by MQO in the physiology of *C. glutamicum* needs to be emphasized. MQO has dual roles in both carbon and energy metabolism (Molenaar et al., 1998, Molenaar et al., 2000, Nantapong et al., 2004); MQO works as a TCA cycle enzyme catalyzing the conversion of malate to OAA, a key metabolite connecting catabolism and anabolism. Moreover, MQO constitutes a unique shuttle reaction that re-oxidizes NADH in a coupling reaction with MDH, through which electrons are transferred from NADH to the respiratory chain. In this MQO/MDH coupling system, MDH oxidizes NADH to reduce OAA to malate and then MQO oxidizes malate to OAA with menaquinone as an electron acceptor. Furthermore, among the NADH-oxidizing respiratory enzymes, the MQO/MDH coupling system has been identified as the major primary gate to the respiratory chain, having a large impact on the respiration activity in *C. glutamicum* (Sawada et al., 2012). The importance of MQO was again confirmed in this study; the specific activities of LldD and NDH-II were relatively low as compared to that of MQO in the wild-type strain (Table 3). These facts place MQO as a core enzyme involved multilaterally in *C. glutamicum* metabolism. We showed variation of MQO activities under different metabolic stresses (Table 6). Upregulation of protein synthesis (Li et al., 2007), specific activity and gene expression (Sawada et al., 2012) of both MQO and MDH were observed in the H^+^-ATPase defective mutants of *C. glutamicum* probably to reoxidize NADH formed at a higher rate in the mutants than in the wild-type strains. In this study, to manage metabolic stresses resulting possibly from OAA over-accumulation under biotin-sufficient conditions, strain D1 reduced the MQO activity by down-regulating transcription of *mqo*, which was not observed under biotin-limited conditions (Table 6). In all cases, variations correlated positively with respiratory activity (Table 6). The significant traits of MQO in strain D1 suggest that the reaction catalyzed by MQO is a preferable control point for regulating the intermediate concentration in *C. glutamicum*. From these observations, we propose that MQO functions as a safety valve in fine-tuning the carbon flow around the OAA node and/or controls the cellular redox balance (NAD^+^/NADH) in response to various metabolic constraints in *C. glutamicum*.

**Table 6:** Effect of various metabolic stresses on MQO activity, *mqo* transcriptional level, and respiratory activity.

	Fold change: mutant to wild-type *C. glutamicum* ATCC13032		
	*pyk*-Deleted strain D1		H^+^-ATPase-defective mutant A-1
	Biotin-limited^a^	Biotin-sufficient^b^	Biotin-sufficient^c^
MQO activity	1.1 →	0.37 ↓	2.6 ↑
*mqo* transcriptional level	0.9 →	0.61 ↓	1.4 ↑
Respiratory activity	1.0 →	0.43 ↓	1.4 ↑

Symbols: upward arrow, increased; rightward arrow, no change; downward arrow, decreased.

a Data in Table 2, Table 3, Table 4 obtained from Medium F4 were used.

b Data in Table 2, Table 3, Table 4 obtained from Medium F5 were used.

c Data were according to Sawada et al. (2012).

Although reports regarding the distribution and function of MQO are limited, traits of *mqo*-deletion mutants add insight into the importance of MQO in *C. glutamicum*. An *mqo*-deleted mutant of *C. glutamicum* showed no growth in glucose minimal medium, while the *mdh* (MDH gene)-deleted mutant showed no apparent growth defect, suggesting that MQO has an essential physiological function in this bacterium (Molenaar et al., 2000). *Helicobacter pylori* (Kather et al., 2000) and *Pseudomonas aeruginosa* (Kretzschmar et al., 2002) probably use only MQO for the conversion of malate to OAA, as no MDH activity was detected in *P. aeruginosa*, and no corresponding gene was found in the genome of *H. pylori*. In contrast, *E. coli* seems to depend more on MDH than on MQO for growth. An *mdh*-deleted mutant showed severe growth defects on pyruvate, lactate, malate, succinate or acetate (but not on glucose), whereas an *mqo-*deleted mutant did not (van der Rest et al., 2000). Thus, despite the important roles played by MQO in *C. glutamicum*, the physiological importance of MQO differs in other bacteria.

## 5 Conclusions

We found enhanced biomass production and imbalanced distribution of metabolic intermediates in central metabolic pathways in a *pyk*-deleted mutant of *C. glutamicum* cultured in a complex medium under biotin-sufficient conditions. The activity changes of PEPCK, PEPC, MQO and probably PCx together with both the enhanced glucose consumption and enhanced biomass production were responsible for overcoming the metabolic disturbance caused by the *pyk* deletion. A previous report (Sawada et al., 2012) and this study implicated MQO as the key controlling module in regulation of TCA cycle flux and/or redox balance in this bacterium. These results suggest that *C. glutamicum* has a distinctive strategy for overcoming metabolic disturbance using MQO as the control module. This unique mechanism manifested in *C. glutamicum* may contribute to the sophisticated utilization of this bacterium as a metabolite producer in fermentation industries.

## Footnotes

Appendix A Supplementary data associated with this article can be found in the online version at doi:10.1016/j.meteno.2015.07.001.
